# Effects of an OX2R agonist on migration and removal of tau from mouse brain

**DOI:** 10.1038/s41598-024-64817-8

**Published:** 2024-07-10

**Authors:** Michiko Terada, Kayo Mitsukawa, Masanori Nakakariya, Tatsuki Koike, Haruhide Kimura

**Affiliations:** Takeda Pharmaceutical Company Limited, 26-1, Muraoka-Higashi 2-Chome, Fujisawa, Kanagawa 251-8555 Japan

**Keywords:** Drug discovery, Neuroscience

## Abstract

Pathological proteins including tau are produced in neurons and released into interstitial fluid (ISF) in a neural activity-dependent manner during wakefulness. Pathological proteins in ISF can be removed from the brain via the glymphatic pathway during nighttime. Thus, in individuals with Alzheimer’s disease (AD) that have dysregulated sleep/wake rhythm, application of orexin receptor 2 (OX2R) agonists during daytime could recover the efflux of pathological proteins to ISF and indirectly promote the glymphatic pathway by improving the quality of nighttime sleep after proper daytime arousal, resulting in increased removal of these proteins from the brain. We investigated this hypothesis using OX-201, a novel OX2R-selective agonist with a 50% effective concentration of 8.0 nM. Diurnal rhythm of tau release into hippocampal ISF correlated well with neuronal activity and wakefulness in wild-type mice. In both wild-type and human P301S tau transgenic mice, OX-201 induced wakefulness and promoted tau release into hippocampal ISF. Human P301S tau transgenic mice, tested under our conditions, showed longer wakefulness time, which differs from individuals with AD. OX-201 treatment over 2 months did not alter hippocampal tau levels. Although further studies are required, at a minimum OX2R agonists may not exacerbate tau accumulation in individuals with tauopathy, including AD.

## Introduction

Accumulation of pathological proteins, such as amyloid beta (Aβ) and tau, in the brain is a common pathological feature of Alzheimer’s disease (AD)^1–3^. The possible relationship between Aβ/tau accumulation and dysregulated sleep/wake rhythm has gathered attention. In fact, insomnia, hypersomnia, circadian rhythm sleep disorder, and obstructive sleep apnea are recognized as risk factors for AD^4,5^. Thus, the sleep/wake rhythm might be involved in the production and/or removal of these pathological proteins.

Aβ is generated in neurons through proteolytic processing of the transmembrane amyloid precursor protein (APP) by β- and γ-secretases, where the Aβ peptide is released into the extracellular space^6^. Tau is produced in and released by neurons into the extracellular space and predominantly found in the cytoplasm^7^. Both Aβ and tau aggregation are concentration-dependent processes, where tau aggregation is also associated with abnormal phosphorylation^8–10^. As a result, pathological tau aggregates are predominantly found in the cytoplasm. Thus, release of Aβ/tau into the interstitial system of the brain, filled with interstitial fluid (ISF), could be the first step in controlling accumulation of these proteins. Recent studies suggest that the release of Aβ/tau increases in an excitatory neural activity-dependent manner^9,11^. During wakefulness, when neuronal activity is high, Aβ/tau levels in hippocampal ISF of mice are higher than during the sleep phase^7,9^. In individuals with AD, the release of Aβ/tau into brain ISF may be reduced because tau overexpression suppresses neuronal activity in mice^12^. Thus, stimulating healthy arousal during daytime would be critical to facilitate the release of the pathological proteins into brain ISF in individuals with AD. In line with this hypothesis, excessive daytime sleepiness (EDS) and napping correlated with Aβ deposition in cognitively normal adults^13^. Further, older men taking a nap for ≥ 120 min had 66% higher risk of developing cognitive impairment in 12 years, compared with those who napped for < 30 min per day^13^.

Based on recent findings, the glymphatic pathway is a clearance pathway subserving the para-arterial inflow of cerebrospinal fluid (CSF) into the interstitial system of the brain leading to the cervical lymph nodes via para-venous space and could have a pivotal role in the removal process of the pathological protein aggregates^14–16^. Further, the glymphatic pathway is predominantly active during sleep. One study in rodents found that glymphatic influx increased by 95% and clearance of Aβ in the cortex occurred twice as fast during slow wave sleep, the deepest stage of non-rapid eye movement (NREM) sleep, compared with wakefulness^17^. Thus, high-quality sleep is critical to facilitate the removal of pathological proteins from the brain. Nighttime insomnia often observed in individuals with neurodegenerative diseases may relate to dysfunction of this clearance system. Taken together, disruption of the sleep/wake rhythm may enable accumulation of pathological proteins via dysregulation in two steps: excitatory activity-dependent release from neurons during daytime followed by removal from ISF through the glymphatic pathway during nighttime. Accordingly, restoring this cycle may recover removal of pathological proteins from the brain in individuals with AD.

Orexins exert their biological functions through the activation of two G protein-coupled receptors, orexin receptor 1 (OX1R) and orexin receptor 2 (OX2R)^18^. OX2R knockout mice, but not OX1R knockout mice, show apparent narcolepsy-like symptoms including fragmentation of wakefulness, suggesting that OX2R plays a key role in regulating sleep/wake rhythms^19^. In an autoradiography study using rat brain sections with [^3^H]-EMPA, an OX2R-selective radioligand, we confirmed the widespread distribution of OX2R, including within the hippocampus, where ISF Aβ and tau were monitored^20^. Moreover, we confirmed that our novel OX2R-selective agonist, TAK-925 (danavorexton), produces potent arousal and brain-wide neuronal activation assessed by c-Fos expression analysis^21^. Therefore, OX2R, but not OX1R, could be the key receptor to regulate neural activity-dependent release of pathological proteins.

We hypothesize that application of OX2R agonists during the daytime may increase activity-dependent release of pathological proteins from neurons into brain ISF. Additionally, increased wakefulness by OX2R agonists might improve sleep during the following night, resulting in improved clearance of pathological proteins via the glymphatic pathway. Therefore, in individuals with AD with dysregulated sleep/wake rhythms, application of OX2R-selective agonists during the daytime may improve removal of pathological proteins from the brain. As orexin-A (OX-A) has no selectivity for OX2R and no permeability at the blood–brain barrier (BBB), we will characterize a novel OX2R-selective agonist tool, OX-201, to confirm its longer half-life, which is suitable for preclinical studies in rodents over clinical candidates with very short half-lives. We aim to evaluate the effects of OX-201 on tau levels in hippocampal ISF using microdialysis in both wild-type and human P301S tau transgenic (Tg) mice, a commonly used tauopathy mouse model. Additionally, we plan to investigate whether chronic administration of OX-201 can change tau levels in the hippocampus of human P301S tau Tg mice.

## Results

### Characterization of OX-201

To characterize the biological functions of OX2R in rodents, a BBB penetrable and OX2R-selective agonist with a suitable half-life for preclinical studies is necessary. We obtained an appropriate OX2R agonist tool, OX-201, for these preclinical rodent studies.

**Figure 1:**
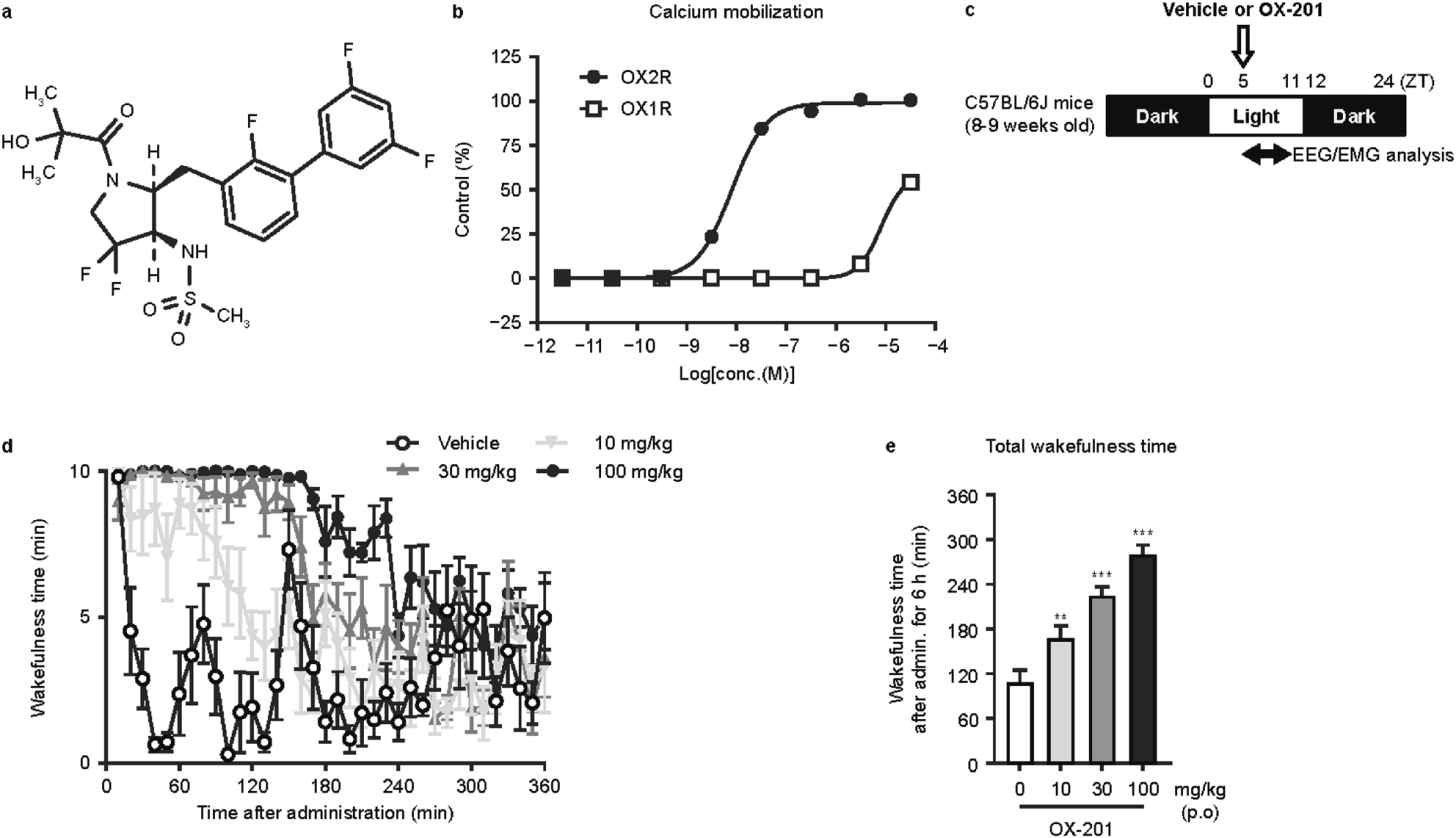
OX-201 selectively activated OX2R and produced wake-promoting effects in mice. (**a**) Chemical structure of OX-201. (**b**) Effects of OX-201 on calcium mobilization in hOX2R/CHO-K1 cells and hOX1R/CHO-K1 cells. The response to 0.5% DMSO in the absence and presence of 100 nM OX-A (positive control) was used to represent the 0% and 100% response, respectively. n = 4. (**c**) Time schedule of drug administration in C57BL/6J mice during the sleep phase. OX-201 was administered orally to mice at ZT5, and then EEG/EMG were recorded. Analysis was performed with data collected for 6 h after administration. (**d**) Effects of OX-201 at 10, 30, and 100 mg/kg on wakefulness time. Mean ± SEM; n = 7. (**e**) Effects of oral administration of OX-201 on total wakefulness time for 6 h after administration in C57BL/6J mice. Mean ± SEM; n = 7; William’s test **p < 0.01, ***p < 0.001 versus vehicle-treated mice. *CHO-K1* Chinese hamster ovary-K1; *DMSO* dimethyl sulfoxide; *EEG* electroencephalogram; *EMG* electromyogram; *h* hours; *hOX1/2R* human OX1/2R; *OX1R* orexin receptor 1; *OX2R* orexin receptor 2; *p.o.* orally; *SEM* standard error of the mean; *ZT* zeitgeber time.

The chemical structure of OX-201 is shown in Fig. 1a^22^. OX-201 showed potent OX2R agonistic activity with 50% effective concentration (EC_50_) of 8.0 nM in a calcium mobilization assay in Chinese hamster ovary-K1 (CHO-K1) cells stably expressing human OX2R (hOX2R/CHO-K1) (Fig. 1b). OX-201 showed very weak OX1R agonistic activity with an EC_50_ of 8.1 µM in a calcium mobilization assay in CHO-K1 cells stably expressing human OX1R (hOX1R/CHO-K1), suggesting 1,000-fold OX2R selectivity over OX1R (Fig. 1b)^22^. In vitro assays for off-target profiling revealed OX-201 at 10 μM did not show > 50% inhibition or stimulation of 102 tested enzymes, receptors, or ion channels, except cannabinoid CB1 receptor (51% inhibition) and progesterone receptor B (53% inhibition), indicating its minimal off-target activity (Supplementary Tables S1 and S2). These results suggest OX-201 is a potent, highly selective OX2R agonist. Oral administration of OX-201 showed a suitable pharmacokinetic profile for in vivo studies in C57BL/6J mice (time to reach maximum concentration [T_max_]: 1–2 h; mean residence time [MRT]: ~ 5 h) (Supplementary Figure S1 and Supplementary Table S3). OX-201 at 10, 30, and 100 mg/kg substantially increased wakefulness time in electroencephalogram/electromyogram (EEG/EMG) recordings during the sleep phase in C57BL/6J mice (Fig. 1c–e).

### Diurnal fluctuation of wakefulness, neuronal activation, and tau release into hippocampal ISF in wild-type mice

**Figure 2:**
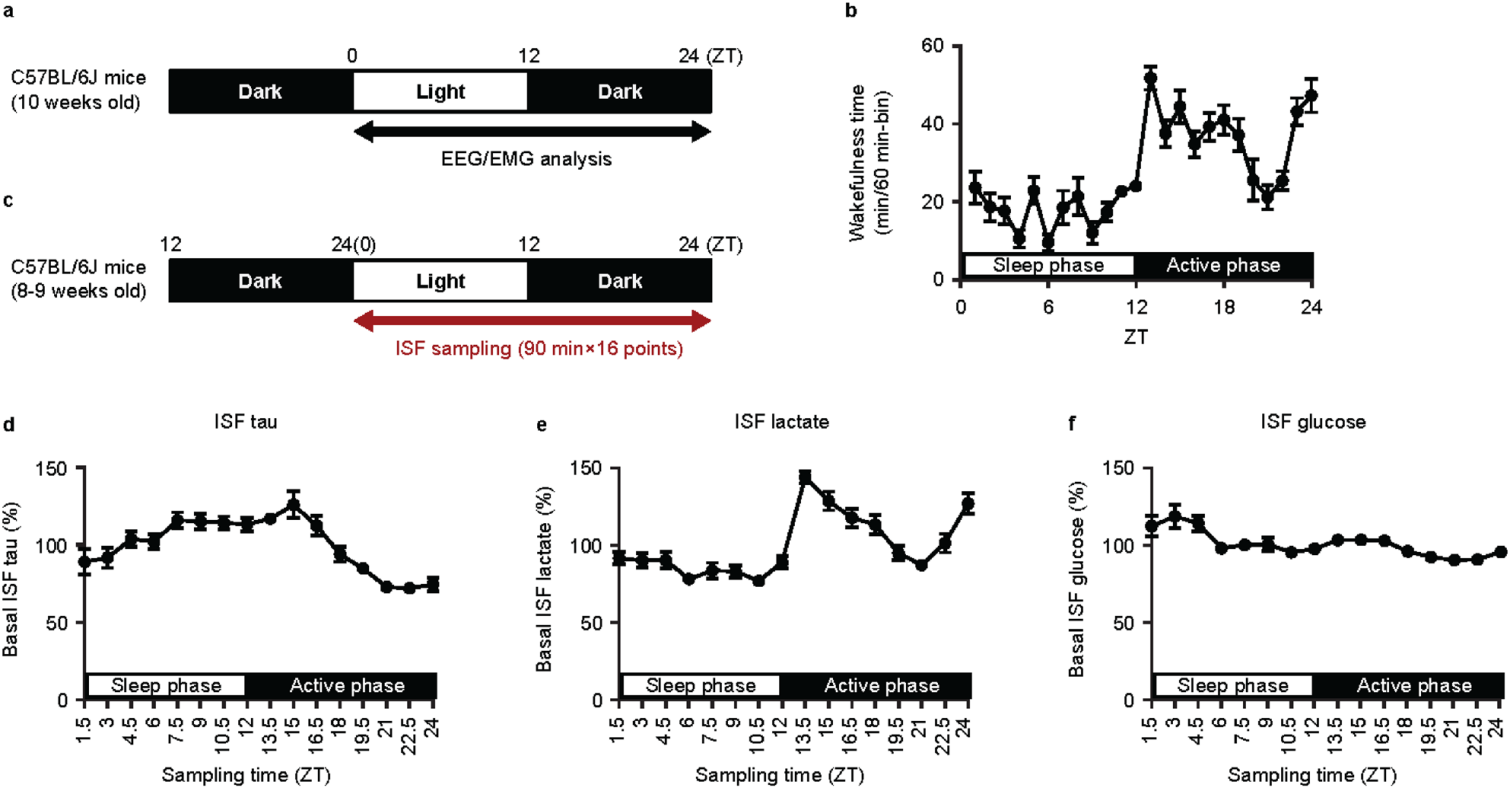
Diurnal fluctuation of wakefulness, neuronal activation, and tau release in hippocampal ISF in wild-type mice. (**a**) Time schedule for EEG/EMG recordings in C57BL/6J mice. (**b**) Sleep/wake rhythm of C57BL/6J mice at 13 weeks old. Mean ± SEM; n = 9. (**c**) Time schedule of ISF sampling using in vivo microdialysis in C57BL/6J mice. The concentration of (**d**) tau, (**e**) lactate, and (**f**) glucose in ISF was expressed as a percentage of the average of the total fraction for 24 h in C57BL/6J mice. Mean ± SEM; n = 6. *EEG* electroencephalogram; *EMG* electromyogram; *ISF* interstitial fluid; *SEM* standard error of the mean; *ZT* zeitgeber time.

First, we assessed wake/sleep rhythms of C57BL/6J mice at 12 weeks old (Fig. 2a). Wakefulness time was longest during the early active phase, decreased into the late active phase, and showed a sharp increase prior to the sleep phase (Fig. 2b).

Next, using in vivo microdialysis, we measured tau levels in hippocampal ISF as well as lactate and glucose levels, which are markers of neuronal activation and brain metabolism, respectively. ISF was collected from freely moving mice implanted with microdialysis probes (Fig. 2c), then concentrations of tau, lactate, and glucose were quantified. Tau levels in hippocampal ISF of C57BL/6J mice showed diurnal rhythm in a similar pattern to the diurnal rhythm of wakefulness (Fig. 2d). Lactate levels also followed the same pattern (Fig. 2e), while no major fluctuations of glucose were observed (Fig. 2f). These results suggest that tau release is closely associated with neuronal activation and wakefulness.

## Effects of OX-201 *on tau* levels in hippocampal ISF of wild-type and human P301S tau Tg mice

**Figure 3:**
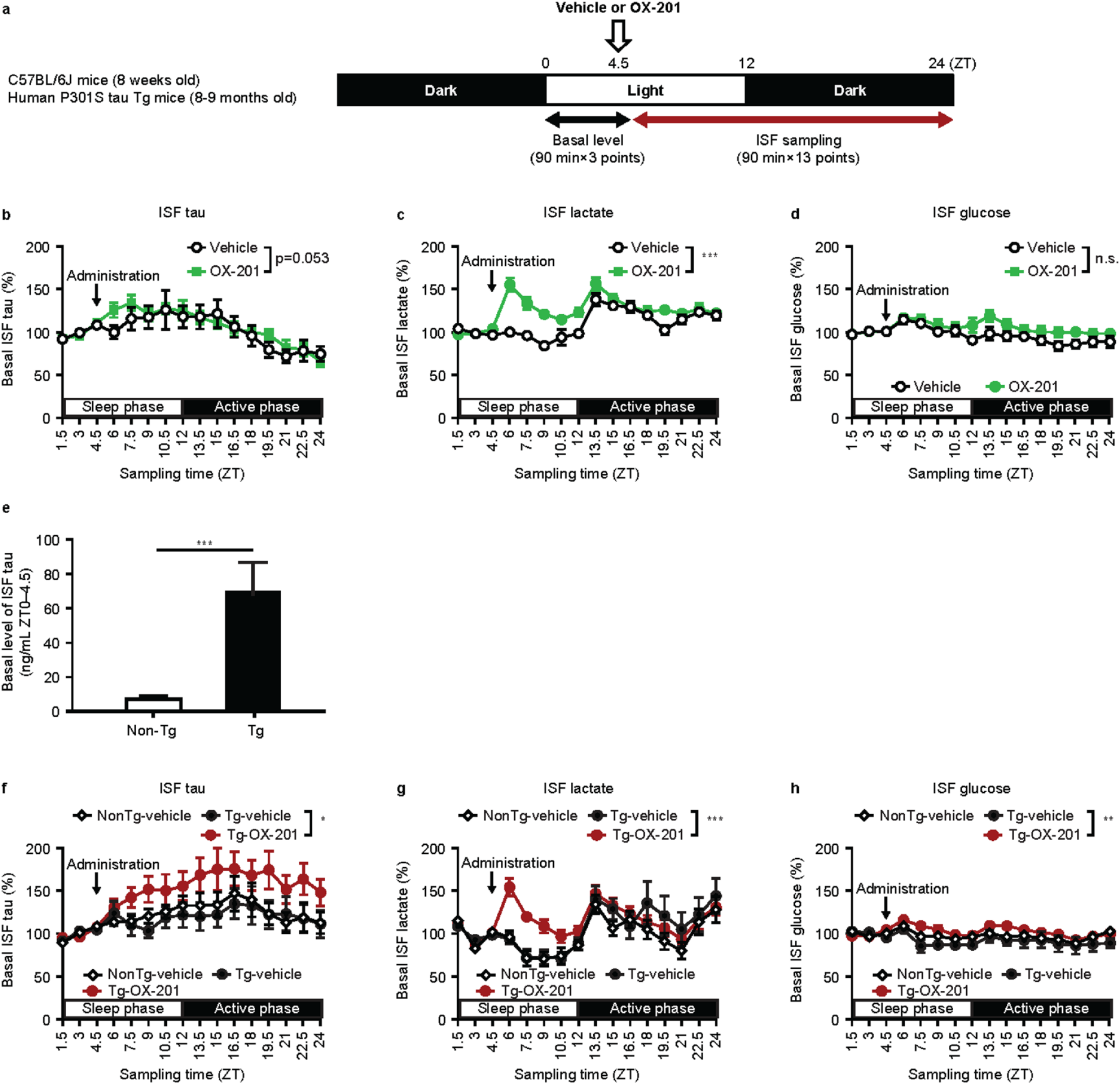
Effects of OX-201 on tau, lactate, and glucose levels in hippocampal ISF of C57BL/6J mice and human P301S tau Tg mice. (**a**) Time schedule for drug administration and ISF sampling in C57BL/6J mice and human P301S tau Tg mice. After baseline ISF collection, vehicle or OX-201 were orally administered at ZT4.5. The effects of OX-201 on (**b**) tau, (**c**) lactate, and (**d**) glucose levels in ISF of C57BL/6J mice. Vehicle-treated mice: n = 5; OX-201-treated mice: n = 8. (**e**) Basal levels of ISF tau were collected for nonTg and human P301S tau Tg mice. NonTg mice: n = 10; Tg mice: n = 21. The effects of OX-201 on (**f**) tau, (**g**) lactate, and (**h**) glucose levels in ISF of human P301S tau Tg mice. Data were shown as a percentage of the basal tau level (average ISF tau levels at ZT0–4.5) in each mouse and shown as a mean ± SEM in each group. Vehicle-treated nonTg mice: n = 10; vehicle-treated Tg mice: n = 9; OX-201-treated Tg mice: n = 12. As the mean residence time of OX-201 was 4.87 h in C57BL/6J mice (Supplementary Figure S1), statistical analysis was performed with the data for 4.5 h after administration of OX-201 using repeated measures ANOVA. *p < 0.05, **p < 0.01, ***p < 0.001. *ISF* interstitial fluid; *SEM* standard error of the mean; *Tg* transgenic; *ZT* zeitgeber time.

To assess the effects of OX-201 on tau release into ISF, levels of tau, lactate, and glucose in hippocampal ISF were measured using in vivo microdialysis after administration of OX-201 at ZT4.5 in C57BL/6J mice at 8 weeks old. The time schedule of drug administration is shown in Fig. 3a. OX-201 at 100 mg/kg was used, which gives potent wake-promoting effects for > 4 h (Fig. 1d). Administration of OX-201 (100 mg/kg) at ZT4.5 showed a tendency to increase hippocampal ISF levels of tau in C57BL/6J mice, where increased tendency was maintained for 4.5 h (Fig. 3b). OX-201 (100 mg/kg) significantly increased hippocampal ISF lactate level, where increased levels were maintained for 7.5 h, while no clear change was observed in hippocampal ISF glucose levels (Fig. 3c, d).

Effects of OX-201 on ISF tau level was also investigated in human P301S tau Tg mice, a tauopathy mouse model with ~ 5 times higher expression of mutant human tau than the endogenous mouse tau^23^. Under our experimental conditions, basal ISF tau levels (average ISF tau levels at ZT0–4.5) of human P301S tau Tg mice at 8–9 months old were ~ 9 times higher than non-transgenic (nonTg) mice (Fig. 3a, e). There was no major difference in the fluctuation pattern of ISF tau levels between human P301S tau Tg mice at 8–9 months old and control nonTg mice as assessed by the percent change from basal level (Fig. 3f). Note that the data were shown as a percentage of the basal tau level (average ISF tau levels at ZT0–4.5) in each mouse. The absolute values of tau in human P301S tau Tg mice are ~ 9 times higher than in nonTg mice as shown in Fig. 3e. As OX-201 at 30 mg/kg already showed considerable wakefulness in C57BL/6J mice, 30 mg/kg of OX-201 was administered to 8–9-month-old human P301S tau Tg mice at ZT4.5 for the microdialysis study. OX-201 at 30 mg/kg increased tau, lactate, and glucose levels in hippocampal ISF of human P301S tau Tg mice (Fig. 3f–h). Higher concentrations of ISF tau were observed > 16.5 h after OX-201 administration (Fig. 3f), while lactate up-regulation was observed for > 6 h (Fig. 3g). These findings indicate OX-201 induced neuronal activation and increased tau levels in ISF in both wild-type and human P301S tau Tg mice. As tau proteins are predominantly cytoplasmic^24^, OX-201 would increase tau levels in ISF by promoting neuronal activity-dependent release of tau from neurons into ISF.

### Characterization of 24-h sleep/wake rhythm in human P301S tau Tg mice

**Figure 4:**
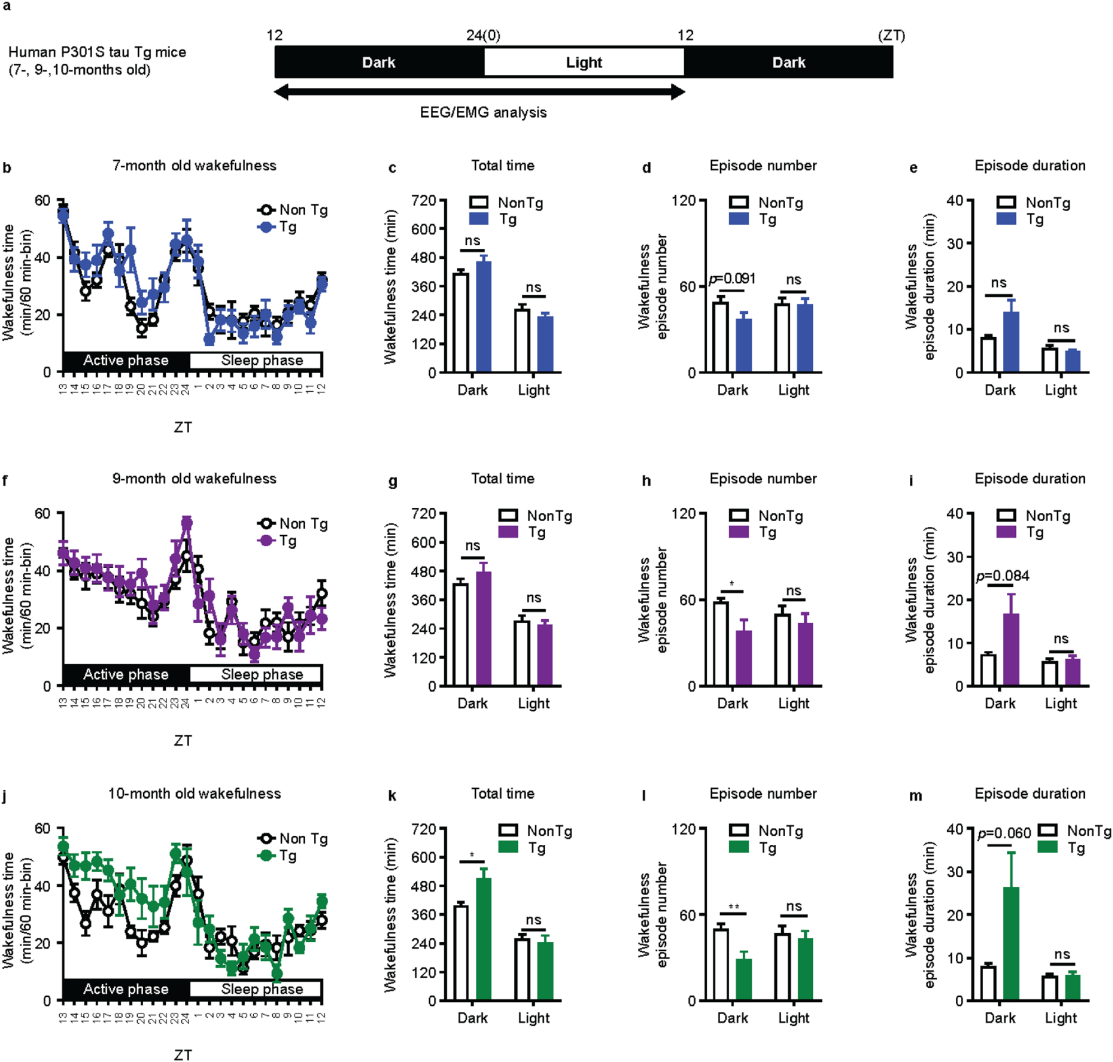
Characterization of the sleep/wake rhythm of human P301S tau Tg mice at 7, 9, and 10 months old. (**a**) Time schedule for EEG/EMG recordings. For human P301S tau Tg mice at 7, 9, and 10 months old, (**b**, **f**, **j**) sleep/wake rhythm, (**c**, **g**, **k**) total wakefulness time, (**d**, **h**, **l**) episode number of wakefulness, and (**e**, **i**, **m**) episode duration of wakefulness were compared with nonTg mice. Mean ± SEM; n = 8. T-test *p < 0.05, **p < 0.01. *EEG* electroencephalogram; *EMG* electromyogram; *ns* not significant; *SEM* standard error of the mean; *Tg* transgenic; *ZT* zeitgeber time.

A recent study showed that individuals with AD have EDS^25^. As human P301S tau Tg mice were reported to show age-dependent sleep impairment in a previous report^26^, we characterized the sleep/wake rhythm of human P301S tau Tg mice by measuring 24-h EEG/EMG at 7, 9, and 10 months old (Fig. 4, Table 1, and Supplementary Figure S2). Human P301S tau Tg mice at 7 months old showed no changes in sleep/wake rhythm compared with nonTg mice (Fig. 4b–e, Table 1, and Supplementary Figure S2). Human P301S tau Tg mice at 9 months old showed considerable decreases in the number of both wakefulness and NREM sleep episodes during the active phase compared with nonTg mice (Fig. 4f-i, Table 1, and Supplementary Figure S2). At 10 months old, human P301S tau Tg mice showed substantial increases in total wakefulness time and substantial decreases in total NREM sleep time during the active phase, accompanied with considerable decreases in the number of both wakefulness and NREM sleep episodes and an upward trend in the duration of wakefulness episodes (Fig. 4j–m, Table 1, and Supplementary Figure S2). These results suggest that human P301S tau Tg mice had higher arousal, not hypersomnolence, during the active phase. These results are consistent with previous reports showing substantial increases in wakefulness time and substantial decreases in both NREM and REM sleep time^26^. Thus, the sleep/wake rhythm in human P301S tau Tg mice differs from the rhythm found in individuals with AD with EDS.

**Table 1:** Changes in sleep/wake rhythms in human P301S tau Tg mice at 7, 9, and 10 months old.

Age	Total time			Episode number			Episode duration		
	Wake	NREM	REM	Wake	NREM	REM	Wake	NREM	REM
Dark period (active phase)									
7 months old	–	–	–	⇣	–	–	–	–	–
9 months old	–	–	–	↓	↓	–	⇡	–	–
10 months old	↑	↓	–	↓	↓	–	⇡	–	–
Light period (sleep phase)									
7 months old	–	–	–	–	–	–	–	–	–
9 months old	–	–	–	–	–	–	–	–	–
10 months old	–	–	↑	–	–	⇡	–	–	–

*Solid arrows indicate significant up-regulation or down-regulation. Dashed arrows indicate a tendency for up-regulation or down-regulation. Wake* wakefulness; *NREM* non-rapid eye movement sleep; *REM* rapid eye movement sleep; *Tg* transgenic.

### Effects of repeated administration of OX-201 on tau accumulation in the *hippocampus* of human P301S tau Tg mice

Human P301S tau Tg mice with hyperarousal during the active phase may not be suitable to evaluate the potential of OX-201 to reduce tau accumulation. However, sleep/wake dysregulation of individuals with AD is heterogenous and understanding the potential risk and benefit of this therapy when used for individuals with AD without hypersomnia is important. Thus, the impact of OX-201 on tau accumulation was assessed in human P301S tau Tg mice.

**Figure 5:**
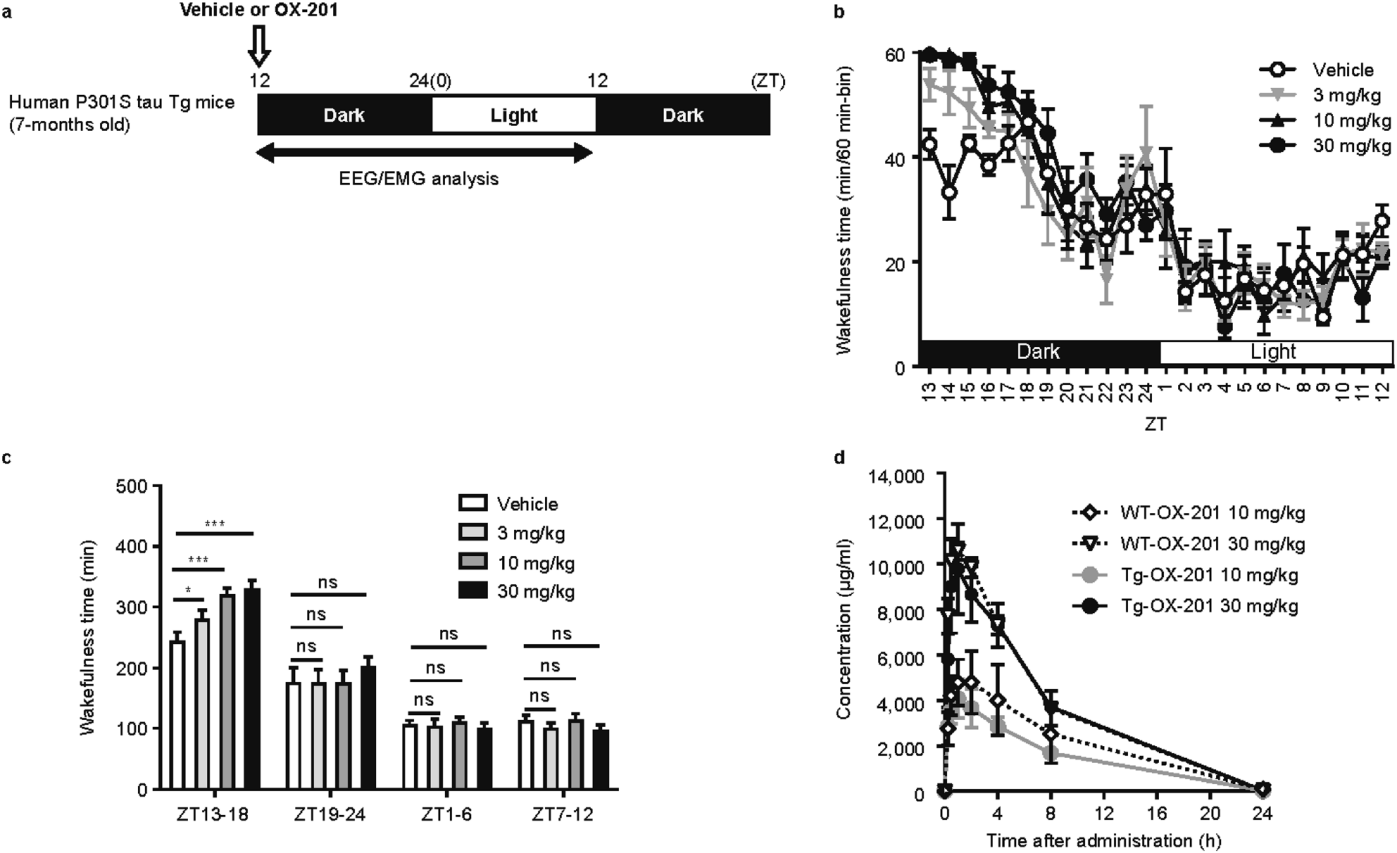
Effects of OX-201 at 3, 10, and 30 mg/kg on wakefulness time in human P301S tau Tg mice at 7 months old. (**a**) Time schedule for drug administration in human P301S tau Tg mice during the active phase. OX-201 was administered orally to mice at ZT12, and then EEG/EMG were recorded. Analysis was performed with data collected during 24 h after administration. (**b**) Effects of OX-201 at 3, 10, and 30 mg/kg on wakefulness time. Mean ± SEM; n = 6. (**c**) Effects of OX-201 on total wakefulness time for 24 h after administration as shown in 6-h groupings. Mean ± SEM; n = 6. William’s test *p < 0.05, ***p < 0.001. (d) Plasma concentrations of OX-201 in human P301S tau Tg mice and nonTg littermates at 7 months old. Mean ± SEM; n = 4. *EEG* electroencephalogram; *EMG* electromyogram; *h* hours; *SEM* standard error of the mean; *Tg* transgenic; *WT* wild type; *ZT* zeitgeber time.

As age-dependent accumulation and phosphorylation of tau in the brain was observed from ≥ 6 months old in human P301S tau Tg mice^23^ and human P301S tau Tg mice began to die at approximately 10 months old, OX-201 was administered for 2 months between 6–8 months old. To determine the dose of OX-201, wake-promoting effects of OX-201 during the active phase were measured in human P301S tau Tg mice at 7 months old by EEG/EMG analysis (Fig. 5a). OX-201 at 3, 10, and 30 mg/kg considerably increased the total wakefulness time for 6 h after administration during the active phase (Fig. 5b, c). Even at the highest dose of 30 mg/kg, OX-201 had no effects on the wakefulness time in the subsequent sleep phase, minimizing the concern of residual wakefulness during the sleep phase. Plasma concentrations of OX-201 showed similar time- and dose-dependent changes between human P301S tau Tg mice and nonTg mice at 7 months old, with undetectable levels at 24 h after administration (Fig. 5d). Thus, progression of tauopathy in human P301S tau Tg mice would not affect the pharmacokinetic profile of OX-201 and consistent exposure of OX-201 would be expected through the 2-month study period.

**Figure 6:**
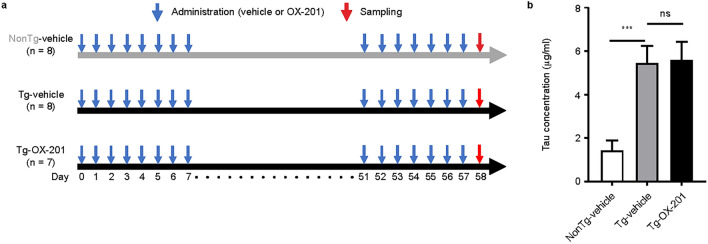
Effect of repeated administration of OX-201 on hippocampal tau levels in human P301S tau Tg mice. (**a**) Time schedule for drug administration in human P301S tau Tg and nonTg littermate mice. OX-201 at 30 mg/kg was administered orally to mice once daily during the active phase (ZT12) for 2 months. (**b**) Effects of repeated administration of OX-201 on tau levels in the hippocampus of human P301S tau Tg mice. Mean ± SEM; n = 7–8. T-test ***p < 0.001. *ns* not significant; *SEM* standard error of the mean; *Tg* transgenic; *ZT* zeitgeber time.

To evaluate the effects of OX-201 on hippocampal tau levels, OX-201 at 30 mg/kg was administered once daily to human P301S tau Tg mice during the active phase (ZT12) for 2 months between 6–8 months old (Fig. 6a). Repeated administration of OX-201 at 30 mg/kg did not alter hippocampal tau levels in human P301S tau Tg mice (Fig. 6b), suggesting that OX-201 had no substantial effects on tau accumulation in the hippocampus.

## Discussion

We hypothesized that disruption of the sleep/wake rhythm results in accumulation of pathological proteins in two steps: dysregulated neural activity-dependent release from neurons into ISF during the active phase and impaired removal from ISF through the glymphatic pathway during the sleep phase.

OX-A and orexin B are neuropeptides released from lateral hypothalamic orexin neurons that regulate multiple physiological functions including wakefulness by neuronal excitation^19,27^. As conventional radioimmunoassay methods to measure OX-A have poor selectivity for OX-A, we established a novel, accurate method using nanoflow liquid chromatography–high-resolution mass spectrometry (nanoLC-HRMS)^28^. Evaluation of diurnal fluctuation of OX-A in monkey cisternal CSF using this method revealed that OX-A levels gradually increased during the active period, reached a maximum at the end of the active period, then dropped to the trough level of the sleep period within 2 h^28^. Although the sleep/wake structure of monkeys and rodents is different (monkeys have a monophasic structure while rodents have a polyphasic structure), the diurnal fluctuation pattern of OX-A may be similar to that of Aβ^9^. Moreover, intracerebroventricular injection of OX-A increased ISF Aβ levels, whereas intracerebroventricular administration of almorexant, a dual orexin receptor antagonist, abolished the diurnal fluctuation of Aβ in mutant human APP-overexpressing mice (Tg2576 mice)^9^. In addition, sleep deprivation can increase OX-A levels in squirrel monkeys^20^ and activate neural activity-dependent release of tau in mice^7^. Therefore, orexins may have a pivotal role in the regulation of neural activity-dependent release of pathological proteins.

It was challenging to test our hypothesis using OX-A due to a lack of selectivity for OX2R over OX1R and no BBB permeability. Thus, a novel, orally available OX2R-selective agonist with a relatively longer half-life in mice, OX-201, was selected for use in this study. OX-201 had an EC_50_ of 8.0 nM with 1,000-fold selectivity for OX2R over OX1R in a calcium mobilization assay and induced considerable wakefulness in mice with ~ 5 h of MRT.

The diurnal fluctuation pattern of tau in ISF was similar to lactate in ISF and wakefulness time; thus, tau release would be regulated by neuronal activity associated with wakefulness in mice. OX-201 treatment increased tau, lactate, and glucose levels in hippocampal ISF in both wild-type and human P301S tau Tg mice. Since OX-201 could induce neuronal activation and wakefulness, OX-201 was found to increase ISF tau levels via promoting the neuronal activity-dependent efflux of tau proteins from intracellular to extracellular space filled with ISF.

The relevance of orexin in the pathology of AD is gathering attention, although there are conflicting results^9,29–31^. In healthy volunteers, suvorexant, which is a dual orexin receptor antagonist, at 10 and 20 mg acutely decreased the ratio of phosphorylated-tau-threonine-181 to unphosphorylated-tau-threonine-181, Aβ38, Aβ40, and Aβ42 in CSF^31^. However, this study did not show the levels of these proteins in ISF or in the brain. As suvorexant at 20 mg is reported to produce residual somnolence the next day^32^, suvorexant at this dose can inhibit neuronal activity and reduce the release of pathological proteins from neurons during the daytime^9^ resulting in the reduction of pathological protein levels in CSF. Individuals with narcolepsy type 1 (NT1) have reduced levels of pathological proteins such as Aβ, tau, and phosphorylated-tau in their CSF, and decreased amyloid deposition on positron emission tomography scans^29^. However, studies with postmortem brain tissues from individuals with NT1 showed that the prevalence of AD in this population was similar to the prevalence in the general population^33^. Loss of orexin neurons in individuals with NT1 may cause both impaired release of pathological proteins from neurons during the daytime and dysregulated clearance from ISF due to impaired sleep during the nighttime, hence, the mixed results could be anticipated.

Novel therapeutics targeting pathological proteins have been intensely developed; multiple antibodies against pathological proteins are currently undergoing clinical studies^34^. As antibodies do not have direct access to intracellular pathological proteins, OX2R agonists would have synergistic effects with these antibodies by promoting neural activity-dependent efflux of pathological proteins from neurons. Moreover, increased wakefulness by an OX2R agonist during the daytime may also improve the quality of sleep the following night^35^ further promoting the removal of pathological proteins via the glymphatic pathway. If this daytime OX2R agonist-induced indirect improvement of nighttime sleep is not active enough to facilitate increased activation of the glymphatic pathway, an additional application of orexin antagonists during the nighttime may be an interesting option. Importantly, prolonged and high level exposure of an OX2R agonist during the sleep phase, which is associated with insomnia, should be avoided, because high-quality sleep is critical to facilitate the removal of pathological proteins from the brain^36^.

In this study, chronic administration of OX-201 did not affect tau accumulation in the hippocampus of human P301S tau Tg mice. One potential explanation for this negative result may be due to the inappropriate sleep/wake rhythm of human P301S tau Tg mice; human P301S tau Tg mice showed increased wakefulness time during the active phase, while individuals with AD show hypersomnolence during the daytime. Increased wakefulness time in human P301S tau Tg mice during the active phase may cause ceiling effects in the removal of tau through promoting neuronal activation/wakefulness, resulting in no substantial changes of tau accumulation in the hippocampus. Another possibility is too high level of tau expression in human P301S tau Tg mice. If these Tg mice show too severe or artificial tau pathology with an enormous amount of tau, the amount of tau released into ISF by OX-201 might be relatively too small and have limited effects on tau accumulation in the hippocampus. In addition, increased wakefulness time in hyperaroused animals may have smaller impacts on tau release compared to that in animals with hypersomnolence. To further evaluate our hypothesis, additional studies using an animal model with hypersomnolence under more physiologically similar conditions would be needed.

A limitation of this study is the lack of evidence for maintained release of tau into ISF after chronic administration of OX-201. In addition, phosphorylated or aggregated tau levels in the hippocampus was not investigated in this study. In future investigation, if reduction of hippocampal tau is observed in another tauopathy animal model with hypersomnolence, detailed examinations, such as tau release into ISF, output of the glymphatic pathway, and abnormal phosphorylation and aggregation of tau proteins in the hippocampus should be investigated after chronic administration of an OX2R-selective agonist. Moreover, it should be considered that rodents, including mice, have a polyphasic sleep/wake structure, while humans have a monophasic sleep/wake structure. Thus, it would be also desirable to use animals with a similar sleep/wake structure to humans.

In conclusion, a novel, orally available OX2R-selective agonist, OX-201, administered at wake-promoting doses increased neuronal activity and release of tau into ISF in the hippocampus of both wild-type and human P301S tau Tg mice. We also showed that chronic OX-201 treatment did not exacerbate tau accumulation in the hippocampus in human P301S tau Tg mice. Considering the difference between these mice and individuals with AD in sleep/wake structure, further studies are required to evaluate if OX2R agonists can decrease tau accumulation in individuals with AD with hypersomnia by promoting neural activity-dependent efflux of tau from neurons. Importantly, our data suggest that an OX2R agonist does not increase tau accumulation in the hippocampus even in tauopathy under conditions of hyperarousal. These results may be helpful in considering the therapeutic strategy of OX2R agonists in a heterogeneous population of individuals with AD.

## Methods

### Chemicals and solutions

OX-201, N-{(2S,3R)-4,4-difluoro-1-(2-hydroxy-2-methylpropanoyl)-2-[(2,3',5'-trifluoro[1,1'-biphenyl]-3-yl)methyl]pyrrolidin-3-yl}methanesulfonamide, was synthesized by Takeda Pharmaceutical Company Limited. OX-A used for calcium mobilization assays was purchased from Peptide Institute Inc. (Osaka, Japan). For in vitro studies, all drugs were dissolved in dimethyl sulfoxide (DMSO) and diluted in each experimental solution. For in vivo studies, OX-201 was suspended in 0.5% (w/v) methylcellulose (MC) in distilled water (Fujifilm Wako Pure Chemical Corporation, Osaka, Japan), then OX-201 was administered orally to mice in a volume of 10 mL/kg of body weight.

### Calcium mobilization assay in human orexin receptor–expressing cells

CHO-K1 cells (CCL-61; ATCC, VA, USA) stably expressing human OX1R or OX2R (hOX1R/CHO-K1 or hOX2R/CHO-K1 cells) were established as described previously^21^. Calcium mobilization in hOX1R/CHO-K1 or hOX2R/CHO-K1 cells was measured using a functional drug screening system/μCELL system (Hamamatsu Photonics, Hamamatsu, Japan) as described previously^21^. The response to 0.5% DMSO in the absence or presence of 100 nM OX-A (positive control) was used as a 0% and 100% response, respectively.

### In vitro assays for off-target profiling

The activity of OX-201 on a panel of 102 receptors, ion channels, and enzymes was evaluated at Eurofins Panlabs Discovery Services Taiwan (Taipei, Taiwan).

### Animals

Male C57BL/6J mice were supplied by CLEA Japan Inc. (Tokyo, Japan). Male human P301S mutant tau (4R1N) Tg mice were developed at the University of Pennsylvania^23^ and introduced to our facility. All mice were housed under a 12 h-light/dark cycle, lights on at 07:00, with ad libitum access to food (CE-2; 3.391 kcal/g of energy, CLEA Japan Inc., Tokyo, Japan) and water. Euthanasia was tolerated under the conditions if the following criteria were observed: more than 20% reduction of body weight, detachment of EEG/EMG electrodes, obvious abnormalities such as seizure. At the end of the study, all mice were sacrificed by carbon dioxide exposure. Animals were maintained and sacrificed according to the guidelines of the Institutional Animal Care and Use Committee (IACUC) of Shonan Health Innovation Park, which is accredited by the Association and Accreditation of Laboratory Animal Care International (AAALAC). All animal experiments were performed in accordance with ARRIVE guidelines.

### Pharmacokinetic study in C57BL/6J mice and human P301S tau Tg mice

The study was performed according to the protocol (protocol number: AU-00040306 and AU-00020637) approved by the Institutional Animal Care and Use Committee of Shonan Health Innovation Park. Blood samples from C57BL/6J mice and human P301S tau Tg mice were collected from the tail vein using a heparinized tube at various time points (0.25, 0.5, 1, 2, 4, 8, and 24 h) after oral administration of OX-201 (10, 30, and 100 mg/kg). Plasma was separated from the blood samples by centrifugation. The drug concentrations in plasma were quantified with high-performance liquid chromatography-tandem mass spectrometry. The lower limit of quantitation was 10 ng/mL. The maximum concentration (C_max_), T_max_, and MRT were calculated.

### In vivo microdialysis study

To assess tau, lactate, and glucose levels in hippocampal ISF from freely moving C57BL/6J mice or human P301S tau Tg mice, in vivo microdialysis with a 1000 kDa molecular weight cut-off probe was performed at Axcelead Drug Discovery Partners Inc. (Kanagawa, Japan) under approval of the Institutional Animal Care and Use Committee of Shonan Health Innovation Park (protocol number: AU-00021258 and AU-00020637). Guide cannulas (CX-I-4, Eicom, Kyoto, Japan) were unilaterally implanted in the hippocampus (AP: − 3.1 mm, ML: − 2.4 mm, DV: − 1.2 mm relative to bregma) under anesthesia of sodium pentobarbital. On the day of the experiment, dialysis probes (AtmosLM, Eicom) with a 3 mm membrane length were inserted through the guide cannula. A 4% bovine serum albumin/Ringer's solution was used as the perfusion buffer. Before microdialysis sample collection, a pump was run at 10 µL/min flow rate for 3 h. ISF samples from wild-type mice and human P301S tau Tg mice were collected in 90 min fractions at 1 µl/min flow rate. Vehicle or OX-201 was administered at ZT4.5 and a sample collection was performed at ZT4.5–24. ISF samples were collected in a refrigerated fraction collector and analyzed by ELISA. ISF total tau levels were measured by Mouse Total Tau kit (Meso Scale Diagnostics, K151DSD). Lactate levels in ISF were measured by Lactate Assay Kit-WST (DOJINDO, L256). Glucose levels in ISF were measured by Glucose Assay Kit-WST (DOJINDO, #G264). The concentration of each fraction was expressed as a percentage of the average concentration of the three fractions before administration of vehicle or OX-201 (ZT0–4.5), with the basal level being 100%. Following each experiment, dialysis probe placements were verified histologically, and the data were excluded if the membrane portion of the dialysis probe was misplaced.

### EEG/EMG recording in C57BL/6J mice and human P301S tau Tg mice

Implantation of EEG/EMG electrodes and EEG/EMG recordings were performed as described previously^21^. Implantation surgery of EEG/EMG electrodes was performed under anesthesia of sodium pentobarbital or 0.3 mg/kg of medetomidine, 4.0 mg/kg of midazolam, and 5.0 mg/kg of butorphanol in saline according to the protocol (protocol number: AU-00021218 or AU-00040306, respectively) approved by the Institutional Animal Care and Use Committee of Shonan Health Innovation Park. EEG/EMG signals were amplified, filtered (EEG, 0.5–250 Hz; EMG, 16–250 Hz), digitized at a sampling rate of 200 Hz, and recorded using VitalRecorder software (KISSEI COMTEC, Nagano, Japan). Locomotor activity was measured by an infrared activity sensor (Biotex, Kyoto, Japan) and recorded using VitalRecorder software. SleepSign software (KISSEI COMTEC) was used to automatically classify wakefulness, NREM sleep, or REM sleep in 4 s epochs, based on EEG/EMG/locomotor activity data. Each stage was characterized as follows: (1) wakefulness, low-amplitude EEG with high-voltage EMG activities or high-locomotion score; (2) NREM sleep, high-amplitude slow-wave EEG with low-voltage EMG activities; and (3) REM sleep, theta-dominated EEG with EMG atonia. This study was conducted with a crossover design. The time spent in wakefulness, NREM sleep, and REM sleep was calculated.

### Effect of repeat administration of OX-201 on hippocampal tau in human P301S tau Tg mice

The study was performed at Takeda Pharmaceutical Company Limited under approval of the Institutional Animal Care and Use Committee of Shonan Health Innovation Park (protocol number: AU-00021218). In control nonTg mice, vehicle (0.5% MC in distilled water) was administered orally to mice once a day (ZT12) for 2 months. Human P301S tau Tg mice were grouped into two cohorts: a control group and a 2-month treatment group. In the control Tg group, vehicle (0.5% MC in distilled water) was administered orally to mice once a day (ZT12) for 2 months. In the 2-month treatment Tg group, OX-201 (30 mg/kg) was administered orally to mice once a day (ZT12) for 2 months. Tau accumulation was assessed in the hippocampus of human P301S tau Tg mice and nonTg mice. In brief, after treatment with a drug, a mouse was decapitated and the hippocampus was isolated. Then, the hippocampus was homogenized in extraction buffer (5 M guanidine-HCl/50 mM Tris, pH 8.0). The samples were diluted tenfold with cold phosphate-buffered saline including 1X protease inhibitor cocktail and then centrifuged at 16,000 × g for 20 min at 4 °C. Total hippocampal tau levels in human P301S tau Tg mice and control nonTg mice were measured by Mouse Tau (Total) ELISA Kit (Invitrogen, Catalog # KMB7011).

### Statistics

All data were presented as mean ± standard error of the mean. Statistical analyses were performed using EXSUS software (CAC EXICARE Corporation, Tokyo, Japan). For comparisons between two groups, data were analyzed with a two-tailed Student’s t-test for parametric data or a two-tailed Aspin-Welch test for non-parametric data. In dose–response studies, a two-tailed Williams’ test for parametric data or two-tailed Shirley-Williams test for non-parametric data was performed to compare multiple doses of the test compound to the control group. Multiple comparisons among groups were performed using closed testing procedures: two-tailed Student’s t-test for parametric data or two-tailed Aspin-Welch t-test for non-parametric data between nonTg mice administered with vehicle and Tg mice administered with vehicle, followed by those between Tg mice administered with vehicle and Tg mice administered with OX-201. Repeated measures ANOVA was used to analyze time × group interaction. A *p*-value < 0.05 was considered significant.

### Supplementary Information

Supplementary Information.

## Data Availability

The datasets and materials supporting the findings of this study are available from the corresponding author on reasonable request.
