# Supplementary material for: Effects of an OX2R agonist on migration and removal of tau from mouse brain
Source: Sci Rep. 2024 Jul 10;14:15964. doi: 10.1038/s41598-024-64817-8 (PMC11237063; doi:10.1038/s41598-024-64817-8)
Supplement: Supplementary file 1 — Supplementary Information. [file 41598_2024_64817_MOESM1_ESM.docx]

**Supplementary Information**

**Effects of an OX2R agonist on migration and removal of tau from mouse brain**

Michiko Terada, Kayo Mitsukawa, Masanori Nakakariya, Tatsuki Koike, & Haruhide Kimura*

*Corresponding author

**Table of Contents**

**Supplementary Table S1……………………………………………………………………... 2**

**Supplementary Table S2……………………………………………………………………... 4**

**Supplementary Table S3……………………………………………………………………... 7**

**Supplementary Figure S1……………………………………………………………………. 8**

**Supplementary Figure S2……………………………………………………………………. 9**

**Supplementary Tables**

**Supplementary Table S1**. Percent inhibition of enzymes by OX-201 at 10 μM (Eurofins Panlabs Taiwan, Ltd.).

| **Enzyme** | **% inhibition** |
| --- | --- |
| 5-Lipoxygenase | −2 |
| ATPase, Ca2+, skeletal muscle | 18 |
| ATPase, Na+/K+, heart | 13 |
| Carbonic anhydrase II | 14 |
| Catechol-O-methyl transferase (COMT) | −1 |
| Cholinesterase, acetyl | 5 |
| Cyclooxygenase (COX)-1 | 15 |
| Cyclooxygenase (COX)-2 | −1 |
| HMG-CoA reductase | 9 |
| Monoamine oxidase (MAO)-A | 4 |
| Monoamine oxidase (MAO)-B | −5 |
| Nitric oxide synthase, neuronal (nNOS) | 18 |
| Nitric oxide synthase, inducible (iNOS) | −1 |
| Peptidase, factor Xa | −3 |
| Peptidase, matrix metalloproteinase-1 (MMP-1) | −2 |
| Peptidase, matrix metalloproteinase-7 (MMP-7) | −3 |
| Peptidase, matrix metalloproteinase-13 (MMP-13) | −1 |
| Peptidase, metalloproteinase, neutral endopeptidase | 2 |
| Phosphodiesterase (PDE) 10A2 | −1 |
| Phosphodiesterase (PDE) 3 | 6 |
| Phosphodiesterase (PDE) 4D2 | 5 |
| Phosphodiesterase (PDE) 5 | −14 |
| Phosphodiesterase (PDE) 6 | −6 |
| Protein serine/threonine kinase, PRKACA (PKA) | −8 |
| Protein serine/threonine kinase, PRKCA (PKCα) | −10 |
| Protein serine/threonine kinase, ROCK1 | 14 |
| Protein tyrosine kinase, EGF receptor | 18 |
| Steroid 5α-reductase | 5 |
| Xanthine oxidase | 2 |

*EGF* epidermal growth factor; *HMG-CoA* 3-hydroxy-3-methyl-glutaryl coenzyme A*; ROCK1* rho-associated, coiled-coil-containing protein kinase 1. Yamada, R., Koike, T., Nakakariya, M., Kimura, H. Orexin receptor 2 agonist activates diaphragm and genioglossus muscle through stimulating inspiratory neurons in the pre-Bötzinger complex, and phrenic and hypoglossal motoneurons in rodents. *PLOS ONE*. In press. (2024).

**Supplementary Table S2**. Percent inhibition of various receptors or ion channels by OX-201 at 10 µM.

| **Receptors or ion channels** | **% inhibition** |
| --- | --- |
| Adenosine A1 | −2 |
| Adenosine A2A | 6 |
| Adenosine A2B | −14 |
| Adrenergic α1, non-selective | −9 |
| Adrenergic α2, non-selective | 4 |
| Adrenergic β1 | −13 |
| Adrenergic β2 | 7 |
| Adrenergic β3 | 12 |
| Androgen (testosterone) | −13 |
| Angiotensin AT1 | 4 |
| Angiotensin AT2 | 11 |
| Bradykinin B1 | 3 |
| Bradykinin B2 | 12 |
| Calcium channel L-type, benzothiazepine | 29 |
| Calcium channel L-type, dihydropyridine | 27 |
| Calcium channel L-type, phenylalkylamine | 20 |
| Calcium channel N-type | −9 |
| Cannabinoid CB1 | 51 |
| Cannabinoid CB2 | 14 |
| Cholecystokinin CCK1 (CCKA) | −3 |
| Cholecystokinin CCK2 (CCKB) | 8 |
| Dopamine D1 | 14 |
| Dopamine D2L | −6 |
| Dopamine D2S | 6 |
| Dopamine D3 | 2 |
| Dopamine D4.4 | −3 |
| Endothelin ETA | 27 |
| Estrogen receptor (non-selective) | −13 |
| GABAA, chloride channel, TBOB | −7 |
| GABAA, flunitrazepam, central | −9 |
| GABAA, muscimol, central | −14 |
| Glucocorticoid | 9 |
| Glutamate, AMPA | 22 |
| Glutamate, kainate | 2 |
| Glutamate, NMDA, agonism | 5 |
| Glutamate, NMDA, glycine | −17 |
| Glutamate, NMDA, phencyclidine | −4 |
| Glycine, strychnine-sensitive | −9 |
| Growth hormone secretagogue (GHS, Ghrelin) | 10 |
| Histamine H1 | 5 |
| Histamine H2 | −20 |
| Imidazoline I2, central | −13 |
| Insulin | −12 |
| IP (PGI2) | −3 |
| Melatonin MT1 | 21 |
| Muscarinic M1 | −6 |
| Muscarinic M2 | 5 |
| Muscarinic M3 | 9 |
| Nicotinic acetylcholine α3β4 | −4 |
| Opiate δ1 (OP1, DOP) | 7 |
| Opiate κ (OP2, KOP) | −6 |
| Opiate μ (OP3, MOP) | −2 |
| Potassium channel [KATP] | −2 |
| Potassium channel [SKCA] | 12 |
| Progesterone PR-B | 53 |
| Serotonin (5-hydroxytryptamine) 5-HT1A | 10 |
| Serotonin (5-hydroxytryptamine) 5-HT2A | 6 |
| Serotonin (5-hydroxytryptamine) 5-HT2B | 25 |
| Serotonin (5-hydroxytryptamine) 5-HT2C | 23 |
| Serotonin (5-hydroxytryptamine) 5-HT3 | −6 |
| Serotonin (5-hydroxytryptamine) 5-HT4 | 23 |
| Sigma, non-selective | 26 |
| Sodium channel, Site 2 | −3 |
| Tachykinin NK1 | 9 |
| Tachykinin NK2 | −13 |
| Tachykinin NK3 | −1 |
| Transporter, dopamine (DAT) | 6 |
| Transporter, GABA | 3 |
| Transporter, norepinephrine (NET) | 15 |
| Transporter, serotonin (5-hydroxytryptamine) (SERT) | −11 |
| Transporter, vesicular monoamine (non-selective) | 3 |
| Vasopressin V1A | −22 |
| Vasopressin V2 | −1 |

*AMPA* α-amino-3-hydroxy-5-methyl-4-isoxazolepropionic acid; *GABA* gamma-aminobutyric acid; *IP* prostaglandin I2 receptor; *NMDA* N-methyl-D-aspartic acid; *TBOB* t-butylbicycloorthobenzoate. Yamada, R., Koike, T., Nakakariya, M., Kimura, H. Orexin receptor 2 agonist activates diaphragm and genioglossus muscle through stimulating inspiratory neurons in the pre-Bötzinger complex, and phrenic and hypoglossal motoneurons in rodents. *PLOS ONE*. In press. (2024).

**Supplementary Table S3**. Pharmacokinetic parameters of OX-201 in C57BL/6J mice at 30 and 100 mg/kg, orally. Data are expressed as mean; n = 4.

| **Dose** | **PK parameter** | **Value** | **Units** |
| --- | --- | --- | --- |
| 30 mg/kg | C_max_ | 11966.2 | ng/mL |
|  | T_max_ | 1.25 | h |
|  | AUC0-24h | 94021.2 | ng・h/mL |
|  | MRT | 4.87 | h |
| 100 mg/kg | C_max_ | 25028 | ng/mL |
|  | T_max_ | 1.75 | h |
|  | AUC0-24h | 272405 | ng・h/mL |
|  | MRT | 5.75 | h |

*AUC* area under the curve; *C_max_* maximum concentration; *MRT* mean residence time; *PK* pharmacokinetic; *T_max_* time to reach C_max_.

**Supplemental Figures**

**
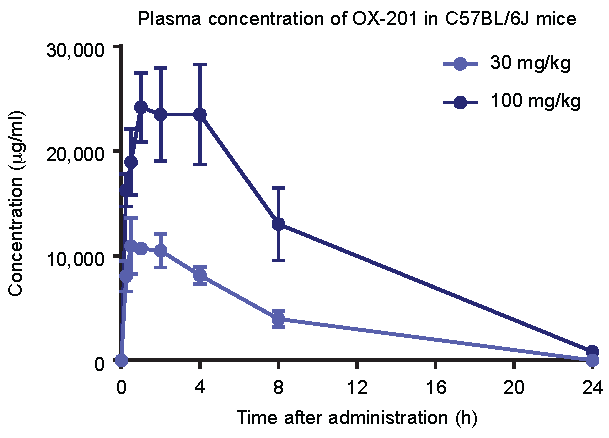
**

**Supplementary Figure S1**. Plasma concentrations of OX-201 (30 and 100 mg/kg) administered orally in C57BL/6J mice at 8 weeks old. Mean ± standard error of the mean; n = 4.


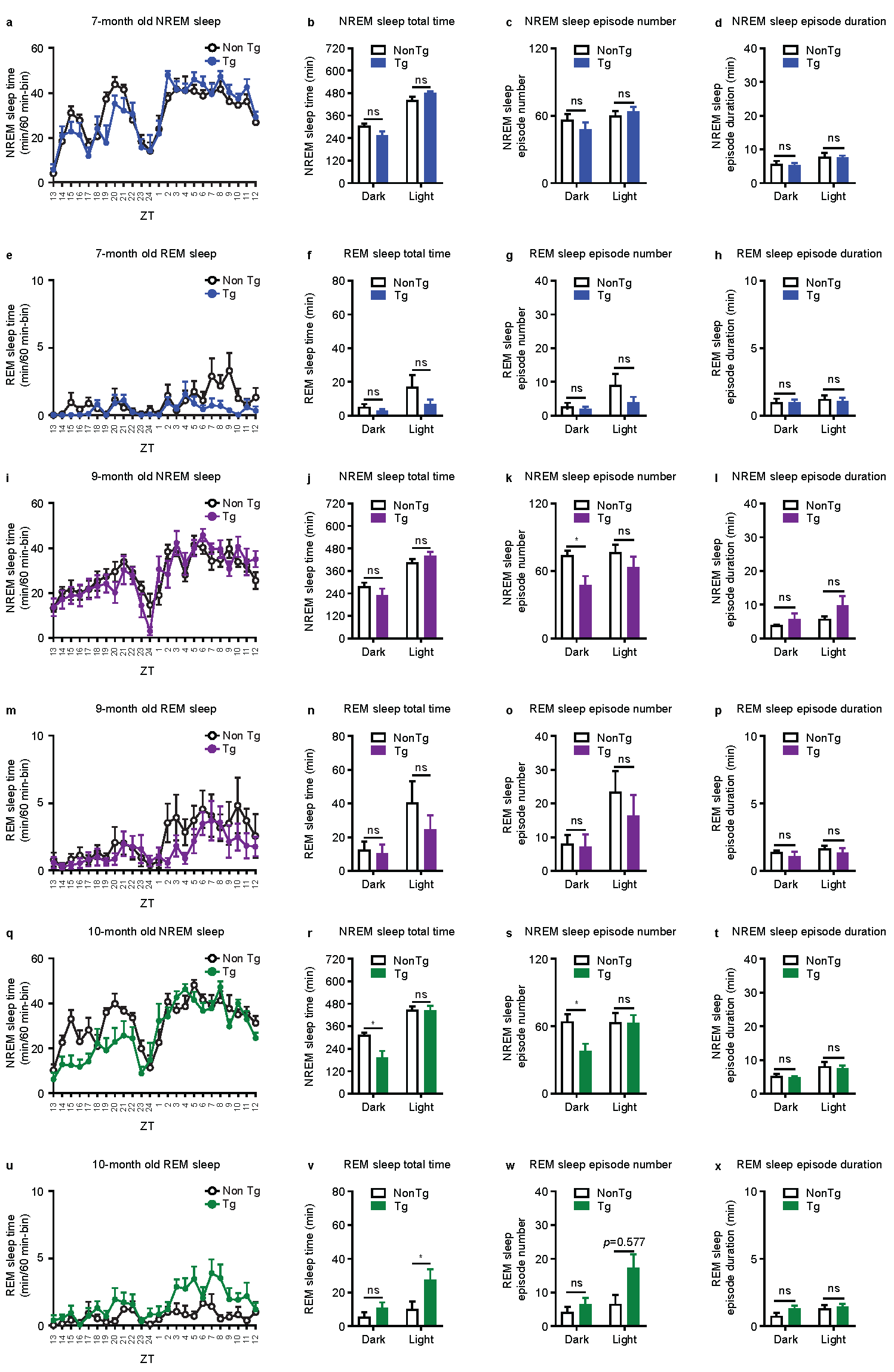


**Supplementary Figure S2**. Characterization of the sleep/wake rhythm of human P301S tau Tg mice at 7, 9, and 10 months old. Temporal pattern of NREM or REM sleep time, total NREM or REM sleep time, episode number, and episode duration in human P301S tau Tg mice at (a-h) 7, (i-p) 9, and (q-x) 10 months old compared with nonTg mice. Mean ± standard error of the mean; n = 8. T-test **p*<0.05.

*ns* not significant; *NREM* non-rapid eye movement; *REM* rapid eye movement; *Tg* transgenic; *ZT* zeitgeber time.
